# Mothers’ experience of maternity and neonatal care when babies die: A quantitative study

**DOI:** 10.1371/journal.pone.0208134

**Published:** 2018-12-05

**Authors:** Maggie Redshaw, Jane Henderson

**Affiliations:** Policy Research Unit in Maternal Health and Care, National Perinatal Epidemiology Unit, Nuffield Department of Population Health, University of Oxford, United Kingdom; University of Liverpool, UNITED KINGDOM

## Abstract

**Background:**

The death of a newborn baby is devastating. While clinical issues may be a primary concern, interpersonal aspects can impact significantly. Mothers in this situation are not easy to access for research and little quantitative evidence is available. In this study we aimed to describe their experience of care, emphasising associations with infant gestational age.

**Methods:**

Secondary analysis of population-based survey data collected through the Office for National Statistics following neonatal death in England in 2012–13. Women were asked about clinical events and care during pregnancy, labour and birth, when the baby died, postnatally and in the neonatal unit.

**Results:**

249 mothers returned completed questionnaires (30% response rate), 50% of births were at 28 weeks’ gestation or less and 66% had babies admitted for neonatal care. 24% of women were left alone and worried during labour and 18% after birth. Only 49% felt sufficiently involved in decision-making at this time. Postnatally only 53% were cared for away from other mothers and babies, 47% could not have their partner stay with them, and 55% were not located close to their baby. Mothers of term babies were significantly less likely to report confidence in staff, feeling listened to and having concerns taken seriously during labour, and postnatally many felt insufficiently informed about their baby’s condition, and that neonatal staff were not always aware of parental needs. However, most mothers (84%) were satisfied with neonatal care.

**Conclusions:**

There is room for improvement if women whose babies die in the neonatal period are to receive the care and support they need. Women who have a baby admitted to a neonatal unit should be cared for nearby, with room for their partner and with greater involvement in decision-making, particularly where withdrawal of life support is considered.

## Introduction

The death of a child is a devastating experience for parents, whether the child is newborn or older, and whether or not the death is expected [1]. While clinical care may be the primary concern, the manner in which care is given can have a huge impact on parental well-being. Where care and follow-up is handled with sensitivity and empathy it may be possible for grieving to begin. However, if parents feel that they were not listened to, were treated dishonestly, without respect, this may lead to complicated grief, severe depression, anxiety and post-traumatic stress symptoms [2, 3].

Qualitative research into parents’ experience of neonatal care has described it as a ‘roller-coaster’ with parents experiencing both joy and grief [4], and both empowerment and powerlessness [2]. Particularly salient themes include parents’ fear of seeing their baby in neonatal care, especially if the baby was very preterm, a fear of getting too close emotionally and of increased grief if the baby should die, the importance of the woman’s partner being able to stay and be included, and the importance of memory making [2]. With parents whose babies are admitted for neonatal care, gestational age and how sick their baby has been can affect how they see their infant and their early parental attachment [5]. Parental expectations and hopes may thus differ if a baby is very preterm, survives labour and is then transferred to neonatal care [6, 7].

A need for effective family-centred care has long been identified [8, 9]. The NHS and other organisations providing support recognise that families may cope better with the stress, anxiety and altered parenting roles in the neonatal unit with family-centred care aimed at facilitating parent-infant attachment and improving long-term outcomes [10]. Parents are thus encouraged to be involved in planning, decision-making and providing care for their baby [11] and if the baby dies, skilled health care professionals need to be available to provide support [10, 12, 13].

For ethical reasons and in recognising the sensitivity of their situation, parents whose babies have died in the neonatal period are relatively rarely included in quantitative studies. This study aimed to better understand their perceptions and experiences. It focuses on care associated with labour and birth, immediate postnatal care and care in the neonatal unit, and how parents’ views and experiences might differ in relation to the gestational age at which their baby was born.

## Methods

This analysis used data collected as part of the Listening to Parents study [14] which employed a similar survey methodology to that used in the earlier National Maternity Surveys. These were cross-sectional surveys using both structured formats and open text questions to investigate women’s experience and perceptions of maternity care during pregnancy, birth and the postnatal period [15–18]. The Listening to Parents survey was carried out in 2012–13 in England. It was developed in association with the stillbirth and neonatal death charities Sands and Bliss in a supportive project advisory group. As with the National Maternity Surveys, the Office for National Statistics (ONS) identified possible participants from birth registrations, in this instance all women aged 16 years and over who registered a stillbirth or neonatal death between January and March 2012 or between June and August 2012 in England. These periods were chosen for pragmatic reasons, to avoid sending questionnaires during holiday periods, whilst avoiding birthdays and anniversaries. The women, 1668 of whom had suffered a stillbirth and 893 whose baby had died as a newborn, were sent an initial letter, followed by a further letter, information sheet and questionnaire (designed either for stillbirth or neonatal death (S1)) between six and nine months after the death. An information sheet in 18 non-English languages gave a Freephone number for contacting the team and getting help in completing the questionnaire, through an interpreter if required. A single reminder was sent to non-respondents after four weeks. Women could call, email or return a blank questionnaire to opt out of the study. In recognition of the potential for distress caused by the survey, the information leaflet gave details of support services offered by Sands, Bliss and other organisations [14]. The research reported here focuses on women’s experiences associated with neonatal death, defined in the UK as a death within the first 28 days after birth. In the UK almost all maternity care is provided by the National Health Service which is free at the point of use.

Structured closed-end questions were used to ask about maternal sociodemographic characteristics, events and care during the pregnancy, at the time of labour and birth, about the baby’s death, and the postnatal period. The range of response options (‘agree’, ‘disagree’ and ‘not sure’ (latter two categories combined); and ‘yes’, ‘to some extent’ and ‘no’ (latter two categories combined) were used in responding to statements about care, so that ‘agree’ and ‘yes’ reflected the best care. ONS provided information about women’s marital status, age group, and Index of Multiple Deprivation in quintiles, an area based measure of deprivation based on income, employment, health and disability [19].

Data arising from responses returned were analysed descriptively using proportions and means or medians as appropriate. Raw percentages, cross-tabulations and chi square statistics were used to test for associations between descriptors of the population and care, and gestational age at birth grouped as follows: less than 28 weeks, 28 to 36 weeks, and 37 or more weeks. Results were considered statistically significant if p was less than 0.05 or 95% confidence interval excluded 1.00. Data were analysed using STATA 13 SE.

Ethics approval was for the study was obtained from National Research Ethics Service Committee South Central–Oxford A on 10^th^ July 2012 (REC Ref. 2/SC/0322). Completion and return of the questionnaire was taken as implicit consent to participate.

## Results

### Sample characteristics

In total, 249 mothers of babies who died in the neonatal period responded, with a usable response rate of 30%. Mothers responding to this survey were more likely to be White, married, UK-born, older, and living in less disadvantaged areas than women with surviving infants who participated in the 2010 National Maternity Survey [17]. Half the babies (50.4%) were born at less than 28 weeks’ gestation, but 29% were born at term (Table 1). Gestational age did not differ significantly with sociodemographic factors, but women with a multiple pregnancy were significantly more likely to deliver preterm. Overall two-thirds of the babies who died were admitted to a neonatal unit but this was significantly less common in very preterm babies. The 84 babies who were not admitted were generally very preterm (61% were less than 25 weeks’ gestation) or had a clinical condition such as a major congenital anomaly.

**Table 1:** Maternal and infant characteristics by gestational age group.

	Gestational age								
	<28 weeks		28–36 weeks		37 or more weeks		Total		p*
*Maternal age*	No.	%	No.	%	No.	%	No.	%	
16–19 years	4	3.4	0	0.0	1	1.4	5	2.1	
20–24 years	20	17.1	7	13.7	7	10.1	34	14.3	
25–29 years	24	20.5	14	27.5	16	23.2	54	22.8	
30–34 years	36	30.8	21	41.2	21	30.4	78	32.9	
35–39 years	24	23.9	9	17.6	20	29.0	57	24.1	
40 or more years	5	4.3	0	0.0	4	5.7	9	3.8	
Total	117	100	51	100.0	69	100	237	100.0	0.607
*Parity*									
Primiparous	59	51.3	24	47.1	42	60.9	125	53.2	
Multiparous	56	48.7	27	52.9	27	39.1	110	46.8	
Total	115	100.0	38	100.0	69	100.0	235	100.0	0.277
*Ethnic group*									
White	93	79.5	42	84.0	57	82.6	192	81.4	
Mixed	0	0.0	0	0.0	3	4.3	3	1.3	
Asian	13	11.1	6	12.0	8	11.6	27	11.4	
Black	9	7.7	1	2.0	1	1.4	11	4.7	
Other	2	1.7	1	2.0	0	0.0	3	1.3	
Total	117	100.0	50	100.0	69	100.0	236	100.0	0.104
Single mother	14	11.5	4	7.8	4	5.8	22	9.1	0.398
*Left full-time education aged…*									
17 or more years	89	77.4	38	74.5	59	86.8	186	79.5	
</ = 16 years	26	22.6	13	25.5	9	13.2	48	20.5	
Total	115	100.0	51	100.0	68	100.0	234	100.0	0.193
*Index of Multiple Deprivation*									
1 (most deprived)	28	23.5	15	30.6	13	19.1	56	23.7	
2	26	21.8	10	20.4	12	17.6	48	20.3	
3	23	19.3	6	12.2	17	25.0	46	19.5	
4	20	16.8	13	26.5	11	16.2	44	18.6	
5 (least deprived)	22	18.5	5	10.2	15	22.1	42	17.8	
Total	119	100.0	49	100.0	68	100.0	236	100.0	0.365
*Multiple pregnancy*									
Singleton	84	68.9	41	80.4	63	92.6	188	78.0	
Twins/triplets	38	31.1	10	19.6	5	7.4	53	22.0	
Total	122	100.0	51	100.0	68	100.0	241	100.0	0.001
Admitted to NNU	51	50.0	44	86.3	51	75.0	146	66.1	<0.001

Numbers of missing values ranged from 0–8 except for Admission to NNU where 21 missing

* Significance of variation by gestational age group

### Clinical aspects of care

Two-thirds of the women in the study (68%) had concerns about their baby prior to labour, commonly due to their gestation but also relating to growth restriction, premature rupture of the membranes or an identified clinical condition such as a heart defect or diaphragmatic hernia (Table 2). Over a third had a hospital admission prior to labour (39%), with nearly half having more than one, most commonly for bleeding. Eighty percent of women had a labour and over half (56%) had a preterm labour, during which a quarter had no electronic fetal monitoring (EFM) of the baby (with over half labouring for less than four hours). Most women (61%) had a normal vaginal delivery and 33% had a Caesarean section, generally due to unforeseen problems in labour, most commonly fetal distress.

**Table 2:** Concerns and clinical events prior to neonatal death.

	Gestational age								
	<28 weeks		28–36 weeks		37 or more weeks		Total		
	No.	%	No.	%	No.	%	No.	%	p*
*Problems with pregnancy or baby*									
High BP/pre-eclampsia	5	4.1	6	11.8	3	4.3	14	5.8	0.120
Bleeding	50	41.0	21	41.2	12	17.4	83	34.3	0.002
Low-lying placenta	11	9.0	7	13.7	1	1.4	19	7.9	0.037
Other placental problem	5	4.1	11	21.6	4	5.8	20	8.3	<0.001
Multiple pregnancy	36	29.5	8	15.7	5	7.2	49	20.2	0.001
Baby not growing well	8	6.6	18	35.3	13	18.8	39	16.1	<0.001
Threatened preterm labour	39	32.0	9	17.6	2	2.9	50	20.7	<0.001
Malformation of the baby	7	5.7	13	25.5	15	21.7	35	14.5	<0.001
Previous CS	13	10.7	6	11.8	2	2.9	21	8.7	0.127
Admission prior to labour	58	50.0	21	43.8	10	15.6	89	39.0	<0.001
>1 admission	22	39.3	13	65.0	3	33.3	38	44.7	0.107
*Concerns about baby before labour*									
Prematurity	69	75.0	23	54.8	2	6.3	94	56.6	<0.001
Growth	8	8.7	17	40.5	10	31.3	35	21.1	<0.001
PROM	33	35.9	6	14.3	3	9.4	42	25.3	0.002
Clinical condition	18	19.6	21	50.0	20	62.5	59	35.5	<0.001
*Laboured*	106	93.8	21	43.8	53	82.8	180	80.0	<0.001
*Monitoring in labour*^*1*^									
No monitoring	36	33.0	1	4.4	5	9.4	46	24.1	<0.001
Intermittent	41	37.6	5	21.7	22	41.5	68	36.8	0.249
Continuous	17	15.6	18	78.3	32	60.4	67	36.2	<0.001
*Mode of delivery*									
SVD	103	87.3	13	25.5	29	42.0	145	60.9	
Instrumental	1	0.8	3	5.9	10	14.5	14	5.9	
Planned CS	6	5.1	19	37.3	7	10.1	32	13.4	
CS due to unforeseen problems	8	6.8	16	31.4	23	33.3	47	19.7	
Total	118	100	51	100	69	100	238	100	<0.001

^1^ Women may have had both intermittent and continuous monitoring at different times

Numbers of missing values ranged from 4–14

* Significance of variation by gestational age group

### Women’s perceptions of their care

Perceptions of intrapartum and immediate postnatal care (Table 3) show that during labour and birth a majority of women felt well cared for in terms of communication, confidence and trust in staff, feeling listened to, their concerns taken seriously and kept informed, although the mothers of term babies were significantly less likely to feel this way. Overall, a lower proportion of women felt that they had a part in decision-making or confident in decisions made and while the majority of women and their partners were not left alone during labour or immediately after birth when it worried them, over a third (38%) were left alone and worried at some point.

**Table 3:** Women's perceptions of their care during labour and birth and in the immediate postnatal period.

	Gestational age								
	<28 weeks		28–36 weeks		37 or more weeks		Total		
*Care during labour and birth*	No.	%	No.	%	No.	%	No.	%	p*
Staff communicated very/quite well	89	76.7	41	80.4	46	68.7	176	75.2	0.298
Always had confidence and trust in staff	72	61.5	35	70.0	30	44.8	137	58.5	0.015
Felt listened to	70	64.9	33	70.2	30	46.2	135	60.5	0.046
Concerns taken seriously	72	64.9	33	73.3	28	42.4	133	59.9	0.006
Informed what was happening	70	61.9	32	68.1	35	53.8	137	60.9	0.519
Had a part in decision-making	54	49.5	24	52.2	31	47.0	109	49.3	0.814
Confident in decisions made	59	52.2	27	58.7	30	46.2	116	51.8	0.391
*Left alone and worried*									
yes, during labour	31	27.0	5	10.2	19	28.8	55	24.0	0.039
yes, shortly after birth	19	16.5	9	18.4	14	21.2	42	18.3	0.734
*Postnatal care*									
Away from other women and babies	64	56.1	21	42.0	24	35.8	109	47.2	0.021
Where partner could stay	65	57.0	21	42.0	36	53.7	122	52.8	0.204
Close to baby	54	47.4	22	44.0	28	41.8	104	45.0	0.757
*Postnatally staff*…									
talked in a way women could understand	105	90.5	42	85.7	56	82.4	203	87.1	0.364
treated women with respect	102	88.7	36	72.0	53	77.9	191	82.0	0.082
treated women with kindness	102	89.5	41	82.0	55	80.9	198	85.3	0.310
listened to women's concerns	91	78.4	35	70.0	48	70.6	174	74.4	0.370

Numbers of missing values ranged from 8–21

* Significance of variation by gestational age group

During the immediate postnatal period when women were still in hospital, although interactions with staff were generally perceived as good, around half of the women reported being cared for in the vicinity of other postnatal women and their babies, especially mothers of term babies, where their partner could not stay with them, and not in a location close to their baby.

### Neonatal care

Of the 146 babies admitted to a neonatal unit, most were preterm or had respiratory problems. Overall, a third of women had met neonatal staff prior to their baby’s admission but significantly more mothers of very preterm babies had done so (Table 4).

**Table 4:** Reasons for admission, duration of stay and mother’s perceptions of care in neonatal unit (NNU) by gestational age group.

	Gestational age								
	<28 weeks		28–36 weeks		37 or more weeks		Total		
	No.	%	No.	%	No.	%	No.	%	p*
*Length of stay in NNU*									
<1 day	10	20.8	17	39.5	15	29.4	42	29.6	
1–6 days	22	45.8	13	30.2	22	43.1	57	40.1	
7 or more days	16	33.3	13	30.2	14	27.4	43	30.3	
Total	48	100.0	43	100.0	51	100.0	142	100.0	0.340
*Reason for admission to NNU*									
Baby premature	50	100.0	33	75.0	2	4.1	85	59.4	<0.001
Breathing problems	14	24.0	25	56.8	31	63.3	70	49.0	0.001
Feeding problems	2	4.0	7	15.9	5	10.2	14	9.8	0.152
Observation only	2	4.0	5	11.4	10	20.4	17	11.9	0.041
Met NNU staff before	24	48.0	18	41.9	10	19.6	52	36.1	0.009
*When baby was in NNU…*									
Equipment always explained	34	69.4	31	70.5	28	59.6	93	66.4	0.389
Procedures always explained	33	68.8	30	68.2	31	66.0	94	67.6	0.873
Baby's problems always discussed	39	79.6	34	77.3	33	67.3	106	74.6	0.298
Baby's treatment always discussed	40	83.3	31	70.5	34	70.8	105	75	0.282
*When baby was in NNU*, *mother able to…*									
Clean baby's face/hands	16	32.7	16	37.2	22	44.9	54	38.3	0.464
Change baby's position	10	20.4	9	20.9	13	26.5	32	22.7	0.694
Change baby's nappy	17	34.7	20	45.5	25	50.0	62	43.4	0.429
Cuddle baby	9	18.4	21	48.8	25	51.0	55	39.0	0.003
*When woman first interacted with baby*									
*Woman first saw baby*…									
at birth	24	49.0	23	53.5	26	52.0	73	51.4	
1st day	24	49.0	18	41.9	21	42.0	63	44.4	
Later	1	2.0	2	4.7	3	6.0	6	4.2	
Total	49	100.0	43	100.0	50	100.0	142	100.0	0.504
*Woman first touched baby…*									
at birth	5	10.6	9	22.0	13	27.1	27	19.9	
1st day	31	66.0	25	61.0	30	62.5	86	63.2	
Later	11	23.4	7	17.1	5	10.4	23	16.9	
Total	47	100.0	41	100.0	48	100.0	136	100.0	0.059
*Woman first held baby…*									
at birth	3	9.1	6	20.0	9	20.5	18	16.8	
1st day	9	27.3	11	36.7	18	40.9	38	35.5	
Later	21	63.6	13	43.3	17	38.6	51	47.7	
Total	33	100.0	25	100.0	44	100.0	107	100.0	0.226
*When baby was in NNU*, *mothers always felt…*									
Supported by staff	38	79.2	36	81.8	33	67.3	107	75.9	0.222
Able to see baby when wished	40	83.3	33	82.5	36	73.5	109	79.6	0.345
Given information about baby's condition	39	81.3	34	77.3	30	60.0	103	72.5	0.024
Staff aware of parental needs	39	81.3	34	81.0	30	61.2	103	74.1	0.038
Involved in decisions about baby's care	30	63.8	30	69.8	31	64.6	91	65.9	0.799
Included in baby's care	30	65.2	29	67.4	29	60.4	88	64.2	0.631
*Satisfaction with neonatal care*									
Satisfied/very satisfied	43	89.6	35	79.6	42	82.4	120	83.9	
Neither satisfied not dissatisfied	5	10.4	6	13.6	7	13.7	18	12.6	
Dissatisfied/very dissatisfied	0	0.0	3	6.8	2	3.2	5	3.5	
Total	48	100.0	44	100.0	51	100.0	143	100.0	0.454

Numbers of missing values ranged from 2–39 (28 women were not well enough to hold their baby)

* Significance of variation by gestational age group

Staff were generally perceived as good at explaining equipment and procedures, discussing the baby’s problems and treatment and giving information about the baby’s condition, but not all women experienced this. Generally, mothers felt well-supported, able to see the baby when they wanted to, and felt staff were aware of their needs, although mothers of term infants were less likely to indicate this. As with intrapartum care, fewer mothers felt involved in decisions about care or included in their baby’s care. Overall satisfaction with neonatal care was very high, 84% of mothers were satisfied (63% ‘very satisfied’) and there were no significant differences by gestational age of the baby in this regard.

Women were asked when they first had contact with their baby. The vast majority (96% and 83%) saw and touched their baby at birth or on the first day. However, it was a while before these mothers were able to hold their sick newborn, and some, though not all did so during the first week if their baby had survived up to then. Similarly, relatively few had been able to provide basic care for their baby, such as changing their baby’s nappy (43%) or cleaning their baby’s face and hands (38%). For these very sick babies, duration of stay in the neonatal unit was generally quite short: 30% stayed less than 24 hours and half of all babies were aged less than 24 hours at death, (65% of those born before 28 weeks). Nearly two thirds of the babies died following withdrawal of life-support. However, only 74% of mothers felt even partly involved in this decision (Table 5). After the baby’s death, almost all mothers saw, held, spent time with, and had photos of their child. Fewer had other children or relatives see the baby, and around half had bathed or dressed their baby. Only 6% took their baby home for a time. Where mothers did not participate in these activities, it was partly due to them not being offered, and partly that the mothers felt they could not or did not want them. Appropriate facilities and information were provided for almost all mothers at this time. The only significant differences by gestational age in these variables were that fewer mothers of term babies had photos, and very few babies of less than 28 weeks’ gestation were taken home at this stage.

**Table 5:** Care and interaction following the baby's death by gestational age group.

	Gestational age								
	<28 weeks		28–36 weeks		37 or more weeks		Total		p*
	No.	%	No.	%	No.	%	No.	%	
*Age at death*									
<1 day	75	65.2	24	47.1	20	30.3	119	51.3	
1–2 days	11	9.6	10	19.6	9	13.6	30	12.9	
3–7 days	14	12.2	7	13.7	16	24.2	37	15.9	
>1 week	15	13.0	10	19.6	21	31.8	46	19.8	
Total	115	100.0	51	100.0	66	100.0	237	100.0	0.001
Life support withdrawn	59	53.2	34	68.0	46	68.7	139	61.0	0.223
Mothers involved to at least some extent in decision	43	65.2	28	75.7	40	87.0	111	73.5	0.121
*After baby's death…*									
Saw baby	116	99.1	46	90.2	65	94.2	227	95.8	0.759
Held baby	111	94.1	46	90.2	63	91.3	220	92.4	0.809
Spent time with baby	114	97.4	43	86.0	63	91.3	220	93.0	0.39
Other children/relatives saw baby	88	78.6	40	78.4	47	69.1	175	75.8	0.316
Had photos of baby	106	90.6	48	94.1	56	81.2	210	88.6	0.033
Dressed baby	60	51.7	35	70.0	37	54.4	132	56.4	0.075
Took baby home for a time	2	1.7	6	12.0	5	7.5	13	5.6	0.001
*Offered…*									
A quiet room to be baby	114	96.6	46	92.0	57	90.5	217	93.9	0.47
Help with funeral arrangements	88	75.2	36	72.0	47	74.6	171	74.3	0.782
Information about support groups	100	85.5	45	90.0	54	85.7	199	86.5	0.976
Written information	103	89.6	38	76.0	54	85.7	195	85.5	0.051
Information about counselling	89	76.1	40	80.0	52	85.2	181	79.4	0.333

Numbers of missing values ranged from 0–14

* Significance of variation by gestational age group

## Discussion

This study has demonstrated that, in the tragic circumstances of their baby dying in the neonatal period, the majority of mothers in this high income country context felt well cared for. However, a small proportion felt they were treated poorly, with a lack of sensitivity. A particular concern was the high proportion of women who were cared for postnatally within sight and sound of healthy mothers and babies and did not have a room where their partner could stay [20]. However, this to some extent mirrors the facilities available for all parents with a baby in neonatal care [8]. The low proportion of mothers who felt able to provide basic care for their baby reflects the fact that these babies were very unwell. Some mothers were able to dress their baby after death, or had mementos such as photos, and where these were not reported it was partly that the mothers felt that they could not or did not want them.

Clinical care for mothers differed little by gestational age of the baby, though those having very preterm babies were significantly less likely to be monitored during labour or to have a Caesarean birth. These very preterm babies were less likely to be admitted to a neonatal unit, but of those babies that were admitted, a higher proportion of mothers than average had met the staff before. Of the mothers whose babies were admitted for neonatal care, those who had term babies that died in the neonatal period did retrospectively report lower levels of confidence and trust in staff at this time, relatively fewer mothers reported always receiving information about their baby’s condition and feeling that staff were aware of their needs as mothers. This parallels an earlier study involving mothers of stillborn babies, nearly one in five of whom felt insufficiently informed about the options for post mortem [21].

Some comparison with a 2014 survey of similar design but targeted at mothers of babies who survived is possible [15]. Mothers whose babies died in the neonatal period were more likely to have a Caesarean birth (33% compared with 26%) and to report being left alone in labour at a time when it worried them (24% compared with 13%) and also afterwards (18% compared with 8%). An almost identical proportion of mothers of babies admitted to a NNU reported that equipment and procedures were explained to them and their baby’s problems discussed. But mothers of surviving babies were able to interact with their babies much sooner (e.g. 81% of mothers of surviving infants admitted to neonatal care saw their baby at birth compared to 51% of mothers whose babies who subsequently died). Similarly, mothers of surviving infants expressed greater confidence and trust in neonatal unit staff (81% compared to 59% respectively), and a higher proportion felt listened to (82% compared to 61% respectively).

This retrospective survey of mothers whose babies had died in the neonatal period was carried out between six and nine months after the event and thus may be subject to recall bias. However, other research on childbirth has confirmed that the salient events at this time are generally well remembered [22, 23]. However, recall at such a stressful time may be affected. The study is limited by the survey response rate of 30% with under-representation of women born outside the UK, those aged less than 30 years, and those living in more deprived areas. Thus the findings may not be representative of the wider population. However, the sample frame is known, was not online or site based [24], and thus the response rate could be calculated and was comparable or better than other similar surveys [25]. Responses were received from 249 women whose baby died in the neonatal period, making it one the largest surveys in this area. Qualitative interview data could have provided a fuller, more in-depth picture of the mothers’ experience of care [4, 7, 26], however this would of necessity have involved a relatively small number of women.

The results are generally consistent with other studies of parents’ experience of stillbirth and perinatal death, including those in countries where resources are more limited [21]. However, circumstances may vary with culture and practice and although in the UK it is recommended [27] that there is a presumption that parents wish to be involved in decisions regarding limiting or withdrawing life support, in this study only 74% of mothers felt involved in the decision at all. A qualitative study of end-of-life decision-making for children found that all parents wanted to be involved but that sometime doctors took over this role [28].

Studies of support for families after perinatal death [29, 30] have highlighted the importance of memory creation, seeing, holding, and taking photos of the baby, in helping families recover. However, there has also been research suggesting that women who held their deceased baby were at greater risk of post-traumatic stress disorder in a subsequent pregnancy [31]. However, qualitative studies, indicate that following perinatal death women welcome and value the opportunity to hold their baby [32].

The emphasis in this study has been on mothers’ experience as the key respondent in the research carried out. The experience of fathers and partners is less commonly the focus in relation to childbirth, stillbirth and neonatal death [24, 33, 34] and yet their care, support role and grief are critical and are a key area for further research.

## Conclusions

In this quantitative study, women who had a baby die in the neonatal period were generally positive about the care they and their baby received. However, there were some differences associated with gestational age and some instances of poor care. In particular, about half of women were cared for in the postnatal period within sight and sound of other women and healthy babies, where their partner could not stay with them, and at some distance from the neonatal unit. This added to the distress felt at an already extremely stressful time. Staff were reported to be kind and respectful but other aspects of care, such as including women in decision-making and women feeling confident in decisions made, were not viewed so positively, particularly during labour and birth. These findings indicate that there is room for improvement if all women whose babies die after birth are to receive the care they need.

## Supporting information

S1 Questionnaire Questionnaire for mothers who had recently experienced neonatal death (NND V3.3). (PDF)

## Abbreviations

BP: blood pressure
CS: Caesarean section
EFM: electronic fetal monitoring
NNU: neonatal unit
SVD: spontaneous vaginal delivery
