# Supplementary material for: Mothers’ experience of maternity and neonatal care when babies die: A quantitative study
Source: PLoS One. 2018 Dec 5;13(12):e0208134. doi: 10.1371/journal.pone.0208134 (PMC6281265; doi:10.1371/journal.pone.0208134)
Supplement: S1 Questionnaire — (PDF) [file pone.0208134.s001.pdf]

Sands
Stillbirth & neonatal death charity

Sands helpline:
020 7436 5881

Bliss
for babies born too soon, too small, too sick

Bliss helpline:
0500 618 140

Listening to Parents

The experiences of women and partners after the death of their baby

We are carrying out this independent survey with the aim of improving care for women who have a baby who dies in the early days or weeks after birth. We feel it is really important to hear the views of women like you who are often not consulted in this way.

We would like to be able to feed back your views about your recent experience to health care providers and the Department of Health who have funded the research. In this way you can help to improve maternity services for women and families who experience such a loss.

The national parent support groups Sands and Bliss are supporting us in this work.

We asked the Office for National Statistics (ONS) to send out this survey on our behalf. They have **not** told us your name and we do **not** have access to any of your details.

If you would prefer not to fill in this questionnaire, please just return it in the pre-paid envelope. This will ensure that we don't contact you again.

If the survey raises issues, emotions or questions of concern, you may wish to contact your family doctor (GP) or Health Visitor or the helplines at Sands or Bliss (see telephone numbers above).

If you would like to talk to someone about this survey, complete the questionnaire over the telephone or with the help of an interpreter, please call us on (Freephone) 0808 252 4566 or email us listeningtoparents@npeu.ox.ac.uk

Maggie Redshaw and Rachel Rowe
National Perinatal Epidemiology Unit
University of Oxford
Old Road Campus
Headington
Oxford OX3 7LF

npeu
National Perinatal Epidemiology Unit

How to complete this questionnaire

- If you have been pregnant before or have experienced the death of a baby before, please only think about the care you received in your recent pregnancy when your baby died when answering these questions.
- For most questions, please tick clearly inside one box ☐ or write clearly on the line or in the box provided. For some questions you may tick more than one box. A small number of questions give you the chance to tell us more about your care and there is a large space at the end where you can tell us anything else that you would like to say about your care.
- The questionnaire is in sections and covers many different aspects of your care during your pregnancy, when your baby died and afterwards. You may not have to complete it all, but you will be guided to the right sections for you. The different sections cover the following topics:
    - Section A: Your baby
    - Section B: Antenatal care
    - Section C: The birth of your baby
    - Section D: If your baby needed specialist care
    - Section E: Your care in hospital after the birth
    - Section F: Your care at home after the birth
    - Section G: Care when your baby died
    - Section H: Father and partner experience
    - Section J: You and your partner's health and wellbeing
    - Section K: Previous and future pregnancies
    - Section L: You and your household
- You may like to take a break while filling in the questionnaire. You may also like to refer to your pregnancy notes, if you still have a copy, for some questions.
- If you have any questions about completing this questionnaire please call us on 0808 252 4566 or email listeningtoparents@npeu.ox.ac.uk

Please tell us the date you filled in the questionnaire D D / M M / Y Y (Day, Month, Year)

Section A: Your baby

A1. Was your baby who died…? ☐1 A single baby ☐2 A twin ☐3 A triplet or more

A2. When was your baby born? D D / M M / Y Y (Day, Month, Year) h h : m m (24hr)

A3. Roughly how many weeks pregnant were you when your baby was born? ☐☐ *weeks*

If you were pregnant with twins, triplets or more and more than one baby died, we may have sent you more than one questionnaire.

If you wish to complete one questionnaire in full for each baby, please do so. Otherwise, please complete one questionnaire in full for your baby who died first, then for your other baby/babies who died please use the other questionnaire(s) and just complete those questions or sections for which your answers are different.

If we have not sent you enough copies of the questionnaire, please contact us.

From here, the questions cover your 'story', from pregnancy to the death of your baby and beyond. In several sections, and at the end, there are boxes for you to say more, in your own words. Please tell us as much or as little as you like.

Section B: Antenatal care

Your early care in pregnancy

B1. Roughly how many weeks pregnant were you when you first realised you might be pregnant? ☐☐ *weeks*

B2. Was this pregnancy the result of infertility treatment? ☐1 Yes ☐2 No

B3. Which health professional did you go to first about your pregnancy care?
Please tick one box only ☐1 Family doctor (GP) ☐2 Midwife ☐3 Other

B4. Roughly how many weeks pregnant were you when you first saw this health professional about your pregnancy care? ☐☐ *weeks*

B5. Roughly how many weeks pregnant were you when you had your 'booking' appointment?
The appointment where you were given your hand held pregnancy records/notes ☐☐ *weeks*

B6. Were any problems identified with you or your baby at or before your antenatal booking? ☐1 Yes ☐2 No

If any problems were identified, what were these?

[ ]

B7. Before you became pregnant did you have any of the following long-term health problems?

Please tick all that apply

	Yes, I experienced this	Yes, I saw a doctor or midwife about this
Diabetes	☐ 1	☐ 2
Anaemia	☐ 1	☐ 2
Epilepsy	☐ 1	☐ 2
High blood pressure	☐ 1	☐ 2
Obesity	☐ 1	☐ 2
Asthma	☐ 1	☐ 2
Anxiety	☐ 1	☐ 2
Depression	☐ 1	☐ 2
Other *Please give details* ____________	☐ 1	☐ 2

If you did have a long-term health problem, did you receive additional or specialist care during your pregnancy for this reason? ☐ 1 Yes ☐ 2 No

If you received specialist care, how well was this co-ordinated?

Please tick one box only ☐ 1 Very well ☐ 2 Quite well ☐ 3 Not very well

Antenatal check-ups

A 'check-up' is any contact with a midwife or a doctor to check the progress of your pregnancy. This usually includes having your blood pressure and urine checked. *Please ignore other appointments that did not include these things, such as a visit* ***only*** *for a scan or a blood test.*

B8. During your pregnancy did you have any antenatal check-ups?

Please tick one box only ☐ 1 Yes ☐ 2 No ☐ 3 Not sure / Don't know

B9. Roughly how many antenatal check-ups did you have from each of the following?

Please write in the number for each

☐☐ Midwife

☐☐ Family doctor (GP)

☐☐ Obstetrician (hospital doctor)

☐☐ Other *Please give details* ____________

B10. During your pregnancy did you have the name and contact details (e.g. phone number) of a midwife or doctor you could get in touch with?

☐ 1 Yes ☐ 2 No ☐ 3 Not sure / Can't remember

B11. During your pregnancy did you ever have an ultrasound scan, showing your baby on a screen? ☐ 1 Yes ☐ 2 No

If No, *please go to B16*

B12. Did you have a dating scan? This takes place between 8 and 14 weeks of pregnancy and tells you when your baby is due.
☐ Yes ☐ No

B13. Did you have a scan at around 20 weeks of pregnancy? This may have been called a '20 week scan', an 'anomaly' scan or a 'mid-trimester' scan.
☐ Yes ☐ No

B14. Roughly how many weeks pregnant were you…
at your first scan? ☐☐ *weeks*
at your last scan? ☐☐ *weeks*

B15. Roughly how many scans did you have in total during your pregnancy? ☐☐ *scans*

B16. Did any of the following affect your pregnancy or your baby? *Please tick all that apply*

	Yes, I experienced this	Yes, I saw a doctor or midwife about this
High blood pressure or pre-eclampsia	☐	☐
Bleeding	☐	☐
Low-lying placenta	☐	☐
Other placental problem	☐	☐
Multiple pregnancy	☐	☐
Baby not growing well	☐	☐
Threatened preterm labour	☐	☐
Malformation of the baby	☐	☐
Previous caesarean (through a cut in your tummy)	☐	☐
Other *Please give details* ________	☐	☐

If you experienced any of the above, did you receive additional or specialist care during your pregnancy for this reason?
☐ Yes ☐ No

If you received specialist care, how well was this co-ordinated?
Please tick one box only
☐ Very well ☐ Quite well ☐ Not very well

Admissions to hospital during your pregnancy

B17. While you were pregnant, before you went into labour did you stay in hospital overnight? *Not counting induction*
☐ Yes ☐ No

If No, *please go to B20*

If Yes, how many times in total? ☐☐ *times*

If Yes, how many nights in total? ☐☐ *nights*

B18. When was this? *Please tick all that apply*

☐ During early pregnancy (1 – 13 weeks)
☐ During mid-pregnancy (14 – 27 weeks)
☐ During late pregnancy (after 27 weeks)

B19. Please tell us the reason(s) for your stay(s) in hospital during your pregnancy?

Your feelings and worries

B20. Thinking about the care you received from staff <u>during your pregnancy</u>, do you agree or disagree with the following statements? *Please tick one box for each line*

Midwives…	Agree	Disagree	Not sure
Talked to me in a way I could understand	☐ 1	☐ 2	☐ 3
Treated me with respect	☐ 1	☐ 2	☐ 3
Treated me with kindness	☐ 1	☐ 2	☐ 3
Listened to my concerns	☐ 1	☐ 2	☐ 3

Doctors…	Agree	Disagree	Not sure
Talked to me in a way I could understand	☐ 1	☐ 2	☐ 3
Treated me with respect	☐ 1	☐ 2	☐ 3
Treated me with kindness	☐ 1	☐ 2	☐ 3
Listened to my concerns	☐ 1	☐ 2	☐ 3

B21. During your pregnancy, <u>before you went into labour</u>, did you have any specific worries about labour and birth? *Please tick one box for each line*

	Very worried	Quite worried	Not very worried	Not at all worried
Not knowing when I would go into labour	☐ 1	☐ 2	☐ 3	☐ 4
Getting to the hospital in time	☐ 1	☐ 2	☐ 3	☐ 4
Having to be induced	☐ 1	☐ 2	☐ 3	☐ 4
Having a long labour	☐ 1	☐ 2	☐ 3	☐ 4
Pain and discomfort of labour	☐ 1	☐ 2	☐ 3	☐ 4
Getting effective pain relief	☐ 1	☐ 2	☐ 3	☐ 4
Not knowing how long labour would take	☐ 1	☐ 2	☐ 3	☐ 4
Having a forceps or ventouse delivery	☐ 1	☐ 2	☐ 3	☐ 4
Embarrassment	☐ 1	☐ 2	☐ 3	☐ 4
Needing a caesarean	☐ 1	☐ 2	☐ 3	☐ 4
That I might die	☐ 1	☐ 2	☐ 3	☐ 4
That my baby might die	☐ 1	☐ 2	☐ 3	☐ 4
Other *Please give details* ________________	☐ 1	☐ 2	☐ 3	☐ 4

B22. Is there anything else you would like to say about your antenatal care?

Section C: The birth of your baby

C1. Were there concerns about your baby before labour and delivery? ☐ Yes ☐ No

If Yes, what were these concerns *Please tick all that apply*

- ☐ My baby was preterm
- ☐ My baby was not growing well
- ☐ My membranes ruptured early
- ☐ My baby had a clinical condition
- ☐ Other *Please give details* ______________________________

C2. Did you have a labour? ☐ Yes ☐ No

If No and you had a caesarean section before labour had started, *please go to C10*

C3. How did your labour start? *Please tick all that apply*

- ☐ It started naturally
- ☐ I was given a vaginal gel or pessary to induce my labour
- ☐ I had one or more membrane sweeps
- ☐ My waters were broken by a doctor or a midwife (amniotomy)
- ☐ I was given a drip (in my hand or arm) to induce my labour

C4. Roughly how long did your labour last? ☐☐ *hours* **and/or** ☐☐ *minutes*

C5. During your labour, which of the following did you use to relieve the pain?
Please tick all that apply

- ☐ Natural methods (e.g. breathing, massage)
- ☐ Water or a birthing pool
- ☐ TENS machine (with pads on your back)
- ☐ Gas and air (breathing through a mask or mouth-piece)
- ☐ Injection of pethidine or a similar painkiller
- ☐ Epidural or spinal (injection in your back)
- ☐ Other *Please give details* ______________________________

C6. During your labour, were you monitored (checked)? *Please tick all that apply*

- ☐ Staff listened with a stethoscope (or ear trumpet) now and then
- ☐ Sonicaid (a hand held monitor) was used now and then
- ☐ A monitor was used now and then, with a belt around my tummy
- ☐ A monitor was used constantly with a belt around my tummy
- ☐ A monitor was used constantly with a clip attached to the baby's head
- ☐ Other *Please give details* ______________________________
- ☐ I had no monitoring
- ☐ Not sure / Can't remember

C7. While you were in labour or before your delivery did you or your partner suspect that something was wrong? ☐1 Yes ☐2 No

If Yes, what made you think there was something wrong? *Please tick all that apply*

- ☐1 The baby's movement slowed or the normal pattern of movement had changed
- ☐1 The baby's heart rate was abnormal
- ☐1 Bleeding
- ☐1 Pain
- ☐1 I just didn't feel things were right
- ☐1 Midwives / doctors were concerned
- ☐1 Other *Please give details* ______________________________

C8. Were you transferred by ambulance during your labour (from home to hospital or between hospitals)? ☐1 Yes ☐2 No

C9. If you were transferred by ambulance...

Roughly how far did you have to travel? ☐☐☐ *miles*

Roughly how long did this take? ☐☐☐ *minutes*

C10. What kind of delivery did you have? *Please tick one box only*

- ☐1 Normal (vaginal) birth
- ☐2 A caesarean (through a cut in your tummy)
- ☐3 Delivery using forceps
- ☐4 Delivery using vacuum cap on the baby's head (ventouse)
- ☐5 Delivery using forceps and vacuum cap (ventouse)

If you had a caesarean, *please go to C12*

C11. If you had a vaginal delivery, did you have any of the following? *Please tick all that apply*

- ☐1 An episiotomy (cut)
- ☐1 A tear (not a deliberate cut)
- ☐1 Stitches
- ☐1 Don't know / Can't remember

If you had a vaginal delivery, *please go to C15*

C12. If you had a caesarean this time was this... *Please tick one box only*

- ☐1 Planned and carried out before you went into labour?
- ☐2 Planned, but carried out after you had gone into labour?
- ☐3 The result of an unforeseen problem during your labour?

C13. For your caesarean, what kind of anaesthetic did you have? *Please tick one box only*

- ☐1 An epidural or spinal anaesthetic
- ☐2 A general anaesthetic (making you go to sleep)

C14. Why did you have a caesarean? *Please tick all that apply*

- ☐1 My baby was 'distressed'
- ☐1 Labour had 'failed to progress'
- ☐1 I wanted my baby to be born in this way
- ☐1 My baby wouldn't fit through my pelvis
- ☐1 Breech presentation (feet or bottom first)
- ☐1 Because I had a caesarean before
- ☐1 I had twins, triplets or more
- ☐1 Because of worries about my health
- ☐1 Because I was in premature labour
- ☐1 Other reason *Please give details:* ______________________
- ☐1 Don't know / Can't remember

C15. Where was your baby born? *Please tick one box only*

- ☐1 In hospital, in a midwife-led unit
- ☐2 In hospital, in a consultant-led unit
- ☐3 In a midwife-led unit or birth centre separate from hospital
- ☐4 At home
- ☐5 Other *Please give details* ______________________

Your care

Please answer the following section if you had a vaginal birth or a caesarean.

C16. Altogether, how many different midwives looked after you during your labour and birth of your baby? *Please tick one box only*

☐1 One ☐2 Two ☐3 Three ☐4 Four ☐5 Five or more

C17. Had you met any of these midwives before? *Please tick one box only*

☐1 All of them ☐2 Some of them ☐3 None of them

C18. Who delivered your baby? *Please tick all that apply*

- ☐1 Midwife
- ☐1 Doctor (obstetrician)
- ☐1 Other *Please give details* ______________________

C19. How well do you feel staff communicated with you about your care in labour and birth?
Please tick one box only

☐1 Very well ☐2 Fairly well ☐3 Not very well ☐4 Not at all well

C20. Did you have confidence and trust in the staff caring for you during your labour and birth?
Please tick one box only

☐1 Always ☐2 Sometimes ☐3 Rarely ☐4 Never

C21. Overall during your labour, did you feel… *Please tick one box for each line*

	Yes	To some extent	No
Listened to?	☐ 1	☐ 2	☐ 3
Your concerns were taken seriously?	☐ 1	☐ 2	☐ 3
Informed about what was happening?	☐ 1	☐ 2	☐ 3
You had a part in decision-making?	☐ 1	☐ 2	☐ 3
Confident about the decisions made?	☐ 1	☐ 2	☐ 3

C22. Were you (and / or your husband, partner or companion) left alone by staff at a time when it worried you? *Please tick one box only*

- ☐ 1 No, not at all
- ☐ 2 Yes, during labour
- ☐ 3 Yes, shortly after the delivery
- ☐ 4 Yes, during labour and shortly after the delivery

C23. Thinking about the care you received from midwives and doctors during your labour and birth, do you agree or disagree with the following statements?

Please tick one box for each line

Midwives…	Agree	Disagree	Not sure
Talked to me in a way I could understand	☐ 1	☐ 2	☐ 3
Treated me with respect	☐ 1	☐ 2	☐ 3
Treated me with kindness	☐ 1	☐ 2	☐ 3
Listened to my concerns	☐ 1	☐ 2	☐ 3

Doctors…	Agree	Disagree	Not sure
Talked to me in a way I could understand	☐ 1	☐ 2	☐ 3
Treated me with respect	☐ 1	☐ 2	☐ 3
Treated me with kindness	☐ 1	☐ 2	☐ 3
Listened to my concerns	☐ 1	☐ 2	☐ 3

C24. We would like to know how you feel you were looked after during your labour and birth. Please circle any of the words below which describe the staff you saw during labour and birth.

Circle as many as you wish

rushed	good-humoured	insensitive	kind
considerate	unhelpful	supportive	offhand
rude	warm	inconsiderate	polite
sensitive	bossy	informative	condescending

Are there any other words you would like to add? ____________________

C25. Is there anything else you would like to say about your care during labour and the birth of your baby?

Section D: If your baby needed specialist care

After birth some babies need specialist care and are admitted to a neonatal unit (NNU), sometimes called a Special Care Baby Unit (SCBU) or Neonatal Intensive Care Unit (NICU).

D1. Was your baby cared for in a neonatal unit at all?
- ☐ 1 Yes
- ☐ 2 No

If your baby was not cared for in a neonatal unit, *please go to Section E*

D2. Why was your baby admitted to a neonatal unit? *Please tick all that apply*
- ☐ 1 My baby was premature
- ☐ 1 My baby had breathing problems
- ☐ 1 My baby had feeding difficulties
- ☐ 1 For observation
- ☐ 1 Other *Please give details* ______________________

D3. Before your baby was born had you met any of the neonatal unit staff?
- ☐ 1 Yes
- ☐ 2 No

D4. Was your baby transferred from one hospital to another?
- ☐ 1 Yes
- ☐ 2 No

D5. If your baby was transferred, why was this? *Please tick all that apply*
- ☐ 1 My hospital did not have a neonatal unit
- ☐ 1 There were no cots available in my hospital
- ☐ 1 My hospital did not have the specialist facilities needed
- ☐ 1 Other *Please give details* ______________________

D6. If your baby was transferred to another hospital, roughly how far away was this?

☐☐☐ *miles*

D7. Was your baby transferred more than once?
- ☐ 1 Yes
- ☐ 2 No
- ☐ 3 Does not apply

D8. How many neonatal units in total provided care for your baby? ☐ *units*

D9. When did you first… *Please tick one box for each line*

	At the birth	First day	First week	More than a week	Was not well enough	Didn't want this
See your baby?	1	2	3	4	5	6
Touch your baby?	1	2	3	4	5	6
Hold your baby?	1	2	3	4	5	6

D10. While your baby was in neonatal care… *Please tick one box for each line*

	Always	Sometimes	Rarely	Never
Was the equipment explained to you?	1	2	3	4
Were the procedures explained to you?	1	2	3	4
Were your baby's problems regularly discussed with you?	1	2	3	4
Was your baby's treatment plan discussed with you?	1	2	3	4

D11. While your baby was in neonatal care did you do any of the following for him or her?
Please tick one box for each line

	Yes	No, my baby was not well enough	No, I was not well enough	No
Help to clean your baby's face or hands	1	2	3	4
Change your baby's position	1	2	3	4
Change your baby's nappy	1	2	3	4
'Top and tail' your baby	1	2	3	4
Cuddle your baby	1	2	3	4
Dress your baby	1	2	3	4
Bath your baby	1	2	3	4
Feed your baby	1	2	3	4

D12. When your baby was in neonatal care, did you feel…

	Always	Sometimes	Rarely	Never
Supported by the staff on the unit?	1	2	3	4
Able to see your baby when you wanted?	1	2	3	4
You were given the information you needed about your baby's condition?	1	2	3	4
That staff were aware of your needs as parents?	1	2	3	4
Involved in decisions about your baby's care?	1	2	3	4
Included in your baby's care?	1	2	3	4

D13. While your baby was in neonatal care did you have any opportunity for skin-to-skin (kangaroo) care? *Please tick one box only*

☐ Quite a lot ☐ Sometimes ☐ Rarely ☐ Not at all

D14. Were you discharged home while your baby was in neonatal care? ☐ Yes ☐ No

D15. During your baby's time in neonatal care did you stay overnight in the hospital?

☐ Yes ☐ No

If Yes, for how many nights in total? ☐☐

D16. If you stayed overnight, where was this? *Please tick all that apply*

☐ A parents' room in or next to the neonatal unit

☐ A parents' room somewhere else

☐ On the postnatal ward

☐ Somewhere your partner could also stay

☐ Other *Please give details* ____________________

D17. For how long was your baby in neonatal care in total?

☐☐ *hours* ***OR*** ☐☐ *days* ***OR*** ☐☐ *weeks*

D18. Overall, how satisfied or dissatisfied were you with the neonatal care your baby received? *Please tick one box only*

☐ Very satisfied ☐ Satisfied ☐ Neither satisfied nor dissatisfied

☐ Dissatisfied ☐ Very dissatisfied

D19. Is there anything else you would like to tell us about your baby's stay in neonatal care?

Section E: Your care in hospital after the birth

This section is about your postnatal maternity unit or hospital stay. If you gave birth to your baby at home and did not spend any time in a maternity unit (or hospital) after the birth *please go to Section F.*

E1. How long did you stay in the maternity unit (or hospital) after your baby was born?

☐☐ *hours* **or** ☐☐ *days*

E2. Where did you stay? *Please tick all that apply*

- ☐ 1 In a single room / private room
- ☐ 1 In a shared room / ward

E3. Where was this? *Please tick all that apply*

- ☐ 1 On the labour ward
- ☐ 1 On the postnatal ward
- ☐ 1 On the antenatal ward
- ☐ 1 High dependency / intensive care
- ☐ 1 Other *Please give details* ____________________

E4. Was this… *Please tick all that apply*

- ☐ 1 Away from other postnatal women and babies?
- ☐ 1 Where your partner could stay?
- ☐ 1 Where you could stay close to your baby?
- ☐ 1 Other *Please give details* ____________________

E5. Thinking about the care you received in the maternity unit (or hospital) after the birth of your baby, do you agree or disagree with the following statements?

Please tick one box for each line

	Agree	Disagree	Not sure
Staff talked to me in a way I could understand	☐ 1	☐ 2	☐ 3
Staff treated me with respect	☐ 1	☐ 2	☐ 3
Staff treated me with kindness	☐ 1	☐ 2	☐ 3
Staff listened to my concerns	☐ 1	☐ 2	☐ 3
Staff treated me as an individual	☐ 1	☐ 2	☐ 3
Staff gave me the care I needed	☐ 1	☐ 2	☐ 3

E6. Looking back, do you feel that the length of your postnatal stay was…

☐1 Too long? ☐2 Too short? ☐3 About right? ☐4 Not sure / Don't know

E7. Is there anything else you would like to say about your postnatal care in the maternity unit (or hospital)? *Please give details*

Section F: Your care at home after the birth

F1. When you were discharged home after the birth of your baby did you have the name and telephone number of a midwife you could contact?

☐1 Yes ☐2 No ☐3 Not sure / Don't know

F2. Were you visited at home by a midwife? *Please tick one box only*

☐1 Yes
☐2 No, I visited the midwife or saw a midwife in a clinic
☐3 No, I was not offered a visit
☐4 No, I was visiting or staying near my baby in a neonatal unit (NNU or SCBU)
☐5 No, I moved house
☐6 No, I did not want a midwife to visit
☐7 No, for another reason

F3. Were you visited at home by your family doctor (GP)?

☐1 Yes ☐2 No ☐3 Not sure / Don't know

F4. Once home, roughly how many times in the first three months did you….

See a midwife at home? ☐☐ *times*
See a maternity support worker at home? ☐☐ *times*
See your family doctor (GP)? ☐☐ *times*
See a health visitor? ☐☐ *times*

F5. How many different midwives in total visited you at home after your baby was born?
Please tick one box only

☐1 None ☐2 One ☐3 Two ☐4 Three or more ☐5 Don't know / Can't remember

F6. Had you met any of these midwives before you had your baby? *Please tick one box only*

☐1 All of them ☐2 Some of them ☐3 None of them ☐4 Not applicable

F7. Did you have confidence and trust in the midwives you saw at home?
Please tick one box only

☐ 1 Always ☐ 2 Sometimes ☐ 3 Rarely ☐ 4 Never ☐ 5 Not applicable

F8. Roughly how long after your baby was born did you have your last visit or contact with a midwife or maternity support worker? *Please tick one box only*

☐ 1 Up to 1 week ☐ 2 2–3 weeks ☐ 3 4–6 weeks ☐ 4 Later than 6 weeks ☐ 5 Not applicable

F9. For postnatal care, would you have liked to have seen a midwife…? *Please tick one box only*

☐ 1 More often ☐ 2 Less often ☐ 3 I saw them as much as I wanted

F10. Is there anything else you would like to say about your postnatal care after your discharge home?

F11. Thinking overall about your care during pregnancy, labour and postnatally, how satisfied or dissatisfied were you with the care you received…
Please tick one box for each line

	Very satisfied	Satisfied	Neither satisfied nor dissatisfied	Dissatisfied	Very dissatisfied
During your pregnancy?	☐ 1	☐ 2	☐ 3	☐ 4	☐ 5
During your labour and delivery?	☐ 1	☐ 2	☐ 3	☐ 4	☐ 5
Postnatally?	☐ 1	☐ 2	☐ 3	☐ 4	☐ 5
After your baby died?	☐ 1	☐ 2	☐ 3	☐ 4	☐ 5

Section G: Care when your baby died

This section of the survey is about what happened around the time that your baby died.

G1. How old was your baby when he or she died?

☐☐ *minutes* **OR** ☐☐ *hours* **OR** ☐☐ *days* **OR** ☐☐ *weeks*

G2. Where did your baby die?

- ☐ On the labour ward / in the operating theatre
- ☐ In the neonatal unit
- ☐ At home
- ☐ Somewhere else *Please give details* ______________________

G3. Were you with your baby when he or she died? ☐ Yes ☐ No

G4. Did your baby die as a result of life support being withdrawn? ☐ Yes ☐ No

If Yes, did you feel involved in decision-making about withdrawal of life support?

☐ Yes ☐ Yes, to some extent ☐ No ☐ Not applicable

G5. After your baby died, did you do the following? *Please tick one box for each line*

	Yes	No, I was not offered this	No, I was not well enough	No, I felt I could not or did not want this
See your baby	☐	☐	☐	☐
Hold your baby	☐	☐	☐	☐
Feel able to spend time with your baby	☐	☐	☐	☐
Have other children or relatives see your baby	☐	☐	☐	☐
Have photos of your baby	☐	☐	☐	☐
Dress your baby	☐	☐	☐	☐
Bath your baby	☐	☐	☐	☐
Have a lock of your baby's hair	☐	☐	☐	☐
Have a copy of your baby's hand or footprints	☐	☐	☐	☐
Take your baby home for a time	☐	☐	☐	☐

G6. If your baby died in hospital were you offered or given any of the following?

Please tick one box for each line

	Yes	No
A quiet room to be with your baby	☐	☐
A blessing or religious ceremony	☐	☐
Help with funeral arrangements	☐	☐
Information about support groups (e.g. Bliss, Sands)	☐	☐
Written information for parents after the death of a baby	☐	☐
Information about counselling services	☐	☐

G7. Have you met with a consultant (senior doctor) to talk over your case, since your baby died? ☐ Yes ☐ No

G8. Have you been given a cause or explanation for your baby's death? *Please tick one box only*

- ☐ Yes, a full explanation
- ☐ Yes, a partial explanation
- ☐ No, I was not given a cause or explanation

G9. Have you been able to ask the questions you wanted? ☐ Yes ☐ To some extent ☐ No

G10. Were you or your partner at any time asked for consent to a post mortem?

☐ Yes ☐ No ☐ No, a post mortem was not necessary

☐ No, the coroner ordered a post mortem

If No, *please go to G12*

If Yes, when were you **first** asked about a post mortem? *Please tick one box only*

- ☐ As soon as my baby's death was confirmed
- ☐ Within 12 hours of my baby's death
- ☐ Within 24 hours of my baby's death
- ☐ More than a day after my baby's death

G11. Who asked you for consent to the post mortem? *Please tick all that apply*

- ☐ Consultant (senior doctor)
- ☐ Junior doctor
- ☐ Midwife
- ☐ Bereavement midwife
- ☐ Don't know / Not sure

G12. Did you… *Please tick one box for each line*

	Yes	No	Not sure	Does not apply
Receive any written information about the post mortem?	☐	☐	☐	☐
Feel you were informed enough to make a choice?	☐	☐	☐	☐
Have enough time to make up your mind?	☐	☐	☐	☐

If you did not have a post mortem, *please go to G16*

G13. What kind of post mortem was carried out?

- ☐ A full post mortem
- ☐ A partial post mortem
- ☐ An external examination of the baby only
- ☐ An examination of the placenta

G14. How long after the post mortem did you find out the results?

- ☐ Within 4 weeks
- ☐ 5 – 8 weeks
- ☐ 9 – 12 weeks
- ☐ After 12 weeks *Please tell us when* ☐☐ *weeks*

G15. How did you find out about the post mortem results? *Please tick all that apply*

- ☐ In a meeting with obstetric / neonatal consultant (senior doctor)
- ☐ In a meeting with bereavement midwife
- ☐ In a meeting with pathologist
- ☐ In an appointment with my family doctor (GP)
- ☐ Other *Please give details* ____________

If you did have a post mortem, *please go to G17*

G16. If you did not have a post mortem, why was this? *Please tick all that apply*

- ☐ The post mortem would take too long
- ☐ The information in the forms was too much to take in
- ☐ Having a post mortem is against my beliefs
- ☐ I didn't need to, as I knew why my baby had died
- ☐ I didn't think it would give us an answer
- ☐ I didn't want my baby's body examined
- ☐ I wasn't offered a post mortem
- ☐ Other *Please give details* ____________

G17. What was the cause or explanation of your baby's death? *Please tick all that apply*

- ☐ My baby had a congenital abnormality
- ☐ My baby's growth was restricted
- ☐ My baby was premature
- ☐ There were problems with my placenta
- ☐ I had a placental abruption (placenta came away before delivery)
- ☐ There were umbilical cord problems
- ☐ My baby died because of trauma during labour
- ☐ My baby died because of lack of oxygen during labour
- ☐ My baby died because of breathing problems
- ☐ My baby died from an infection
- ☐ My baby's life support or care was withdrawn
- ☐ My baby's death was unexplained
- ☐ Other *Please give details* ____________

Section H: Father and partner experience

This section is about your husband or partner's involvement and experience at this difficult time. *If you are without a partner at this time, please answer these questions if your ex-partner or the father of your baby was with you during pregnancy or later, otherwise please go to section J.*

H1. Was your husband or partner present for? *Please tick all that apply*

- ☐1 Your pregnancy test or when your pregnancy was confirmed
- ☐1 One or more of your antenatal checks
- ☐1 One or more of your ultrasound scans
- ☐1 Your labour
- ☐1 The birth of your baby
- ☐1 When your baby was admitted to the neonatal unit
- ☐1 When concerns about your baby were discussed
- ☐1 When your baby died
- ☐1 Not present at all

H2. In general how did your husband or partner feel staff communicated with them?
Please tick one box for each line

	Very well	Quite well	Not very well	Not at all
During pregnancy	☐1	☐2	☐3	☐4
During labour and birth	☐1	☐2	☐3	☐4
After the birth	☐1	☐2	☐3	☐4
When your baby was sick	☐1	☐2	☐3	☐4
When your baby died	☐1	☐2	☐3	☐4

H3. In general did your husband or partner feel… *Please tick one box for each line*

	Yes	To some extent	No
Listened to?	☐1	☐2	☐3
Their concerns were taken seriously?	☐1	☐2	☐3
Informed about what was happening?	☐1	☐2	☐3
They had a part in decision-making?	☐1	☐2	☐3
Confident about the decisions made?	☐1	☐2	☐3
Their needs were acknowledged?	☐1	☐2	☐3

H4. Before your baby died was your husband or partner able to… *Please tick one box for each line*

	Yes	No, they were not offered this	No, they felt they could not or did not want this
See your baby?	☐1	☐2	☐3
Hold your baby?	☐1	☐2	☐3

H5. After your baby died was your husband or partner able to… *Please tick one box for each line*

	Yes	No, they were not offered this	No, they felt they could not or did not want this
See your baby?	☐ 1	☐ 2	☐ 3
Hold your baby?	☐ 1	☐ 2	☐ 3

H6. Was your husband or partner able to take paternity leave when your baby was born?

☐ 1 Yes ☐ 2 No ☐ 3 Does not apply

If Yes, how long was this for? ☐☐ *days* **OR** ☐☐ *weeks*

H7. Was your husband or partner able to take paternity or compassionate leave after your baby died?

☐ 1 Yes ☐ 2 No ☐ 3 Does not apply

If Yes, how long was this for? ☐☐ *days* **OR** ☐☐ *weeks*

Section J: You and your partner's health and wellbeing

J1. Did you have a postnatal check-up of your own health with your GP (family doctor) between 4 and 8 weeks after the birth?

Yes ☐ 1 No ☐ 2

If you did not have a postnatal check-up, why was this? *Please tick one box only*

☐ 1 It was not offered

☐ 2 I did not want to have this check-up

☐ 3 Other reasons *Please give details* ______________________

J2. Did *you* experience any of the following 10 days after the birth of your baby, 3 months after the birth and in the last few days? *Please tick all that apply*

	10 days after the birth	3 months after the birth	In the last few days
Poor physical health	☐ 1	☐ 2	☐ 3
Painful stitches	☐ 1	☐ 2	☐ 3
Breast soreness	☐ 1	☐ 2	☐ 3
Headaches	☐ 1	☐ 2	☐ 3
Crying	☐ 1	☐ 2	☐ 3
Palpitations or feelings of panic	☐ 1	☐ 2	☐ 3
Depression	☐ 1	☐ 2	☐ 3
Wound infection	☐ 1	☐ 2	☐ 3
Leaking urine when you don't mean to (stress incontinence)	☐ 1	☐ 2	☐ 3
Difficulties with eating	☐ 1	☐ 2	☐ 3
Fatigue or severe tiredness	☐ 1	☐ 2	☐ 3

Please continue with this question on the next page

**J2.** Did ***you*** experience any of the following...	10 days after the birth	3 months after the birth	In the last few days
Backache	☐ 1	☐ 2	☐ 3
Anxiety	☐ 1	☐ 2	☐ 3
Difficulties or pain during sexual intercourse	☐ 1	☐ 2	☐ 3
Sleep problems	☐ 1	☐ 2	☐ 3
'Flash-backs' to labour, birth or neonatal unit	☐ 1	☐ 2	☐ 3
Difficulties in concentrating	☐ 1	☐ 2	☐ 3
Not able to go out or leave your home	☐ 1	☐ 2	☐ 3
Difficulties going back to work or at work	☐ 1	☐ 2	☐ 3
Relationship difficulties with your husband or partner	☐ 1	☐ 2	☐ 3
Relationship difficulties with family members	☐ 1	☐ 2	☐ 3
Other *Please give details* ________________	☐ 1	☐ 2	☐ 3

J3. Did *your partner* experience any of the following 10 days after the birth of your baby, 3 months after the birth and in the last few days? *Please tick all that apply*

	10 days after the birth	3 months after the birth	In the last few days
Poor physical health	☐ 1	☐ 2	☐ 3
Headaches	☐ 1	☐ 2	☐ 3
Crying	☐ 1	☐ 2	☐ 3
Palpitations or feelings of panic	☐ 1	☐ 2	☐ 3
Depression	☐ 1	☐ 2	☐ 3
Difficulties with eating	☐ 1	☐ 2	☐ 3
Fatigue or severe tiredness	☐ 1	☐ 2	☐ 3
Anxiety	☐ 1	☐ 2	☐ 3
Difficulties during sexual intercourse	☐ 1	☐ 2	☐ 3
Sleep problems	☐ 1	☐ 2	☐ 3
'Flash-backs' to labour, birth or neonatal unit	☐ 1	☐ 2	☐ 3
Difficulties in concentrating	☐ 1	☐ 2	☐ 3
Not able to go out or leave your home	☐ 1	☐ 2	☐ 3
Difficulties going back to work or at work	☐ 1	☐ 2	☐ 3
Relationship difficulties with you (their partner)	☐ 1	☐ 2	☐ 3
Relationship difficulties with family members	☐ 1	☐ 2	☐ 3
Other *Please give details* ________________	☐ 1	☐ 2	☐ 3

J4. Since your baby died, have you or your partner talked to health professionals or others about what happened and how you feel? ☐1 Yes ☐2 No

If Yes, who have you and your partner talked to? *Please tick all that apply*

	You	Your partner
Midwife who was present during labour or birth	☐1	☐2
Doctor who was present during labour or birth	☐1	☐2
Another doctor not present during labour or birth	☐1	☐2
Nurse from the neonatal unit	☐1	☐2
Doctor from the neonatal unit	☐1	☐2
Family care nurse	☐1	☐2
Bereavement midwife or nurse	☐1	☐2
Family doctor (GP)	☐1	☐2
Health visitor	☐1	☐2
Counsellor	☐1	☐2
Religious advisor	☐1	☐2
Close family and friends	☐1	☐2
Work colleagues	☐1	☐2
Other *Please give details* ____________	☐1	☐2
Don't know	☐1	☐2
Not applicable	☐1	☐2

J5. Who or what has been the most helpful source of support…

For *you*? *Please give details* ____________

For *your partner*? *Please give details* ____________

Section K: Previous and future pregnancies

K1. Do you have plans for future pregnancies? *Please tick one box only*

- ☐1 I am pregnant now ☐☐ *weeks*
- ☐2 I plan to become pregnant in the next few months
- ☐3 I plan to become pregnant, but I'm not sure when
- ☐4 I am not sure about becoming pregnant again
- ☐5 I have decided not to have another baby

K2. Before the pregnancy when your baby died, had you ever been pregnant before? ☐1 Yes ☐2 No

If you have not had any previous pregnancies, *please go to Section L*

K3. Had you given birth before the pregnancy when your baby died? ☐1 Yes ☐2 No

If Yes, how many babies had you given birth to **before** this pregnancy? ☐☐ *babies*

K4. Before your most recent birth had you ever given birth by caesarean section?

☐1 Yes ☐2 No

K5. Have you experienced the loss of a pregnancy or the death of a baby before?

Please tick all that apply

- ☐1 No
- ☐1 Yes – a miscarriage (before 13 weeks)
- ☐1 Yes – a late miscarriage (13 – 24 weeks)
- ☐1 Yes – a termination after 13 weeks
- ☐1 Yes – a stillbirth
- ☐1 Yes – a neonatal death

K6. Before your most recent birth, did you have any long-term health problems which made previous pregnancies difficult or complicated (e.g. diabetes)? ☐1 Yes ☐2 No

Please give details:

K7. Before your most recent birth, did you have problems with other pregnancies (e.g. premature birth, low-lying placenta)? ☐1 Yes ☐2 No

Please give details:

Section L: You and your household

Please answer as many of these questions as you can. This will help us to describe the women taking part in this survey and show whether the care offered to women is the same regardless of their background or circumstances.

L1. How old are you now? ☐☐ *years*

L2. How old were you when you left full-time education?

- ☐1 16 years or less
- ☐2 17 or 18 years
- ☐3 19 years or over
- ☐4 Still in full-time education

L3. Which of the following people live with you? *Please tick all that apply*

- ☐ Other children
- ☐ Husband or male partner
- ☐ Same sex partner
- ☐ Other family members
- ☐ Other people in your household

L4. To which of these ethnic groups would you say you belong? *Please tick one box only*

WHITE

- ☐ 1 British
- ☐ 2 Irish
- ☐ 3 Any other White background

If other, *please write in box:*

BLACK OR BLACK BRITISH

- ☐ 12 Caribbean
- ☐ 13 African
- ☐ 14 Any other Black background

If other, *please write in box:*

MIXED

- ☐ 4 White & Black Caribbean
- ☐ 5 White & Black African
- ☐ 6 White & Asian
- ☐ 7 Any other Mixed background

If other, *please write in box:*

ASIAN OR ASIAN BRITISH

- ☐ 8 Indian
- ☐ 9 Pakistani
- ☐ 10 Bangladeshi
- ☐ 11 Any other Asian background

If other, *please write in box:*

CHINESE OR OTHER ETHNIC GROUP

- ☐ 15 Chinese
- ☐ 16 Any other ethnic group

If other, *please write in box:*

L5. In which country were you born? ______________________

If you were born outside the United Kingdom, what year did you come to the UK? ☐☐☐☐

L6. Do you need any help in understanding English? ☐ 1 Yes ☐ 2 No

L7. Do you have a longstanding physical problem or disability? ☐ 1 Yes ☐ 2 No

L8. Do you have a longstanding mental health problem or learning disability? ☐ 1 Yes ☐ 2 No

L9. Would you mind telling us what your health problem or disability is?

Please give details here: ______________________

L10. Do any of these problems or disabilities affect your day to day activities?

- [ ] Yes, definitely [1]
- [ ] Yes, to some extent [2]
- [ ] No [3]
- [ ] Not applicable [4]

L11. Could you tell us who filled in this questionnaire? *Please tick all that apply*

- [ ] You [1]
- [ ] Your husband / partner [1]
- [ ] Other *Please give details* ____________________ [1]

If there is anything else you would like to tell us about your care while you were pregnant or since your baby died, please add your comments here:

Was there anything else you meant to go back and complete later?

Please check you haven't accidentally missed any pages.

If the survey has raised issues or questions of concern you may wish to contact your family doctor (GP), health visitor, the Bliss helpline (0500 618 140), the Sands helpline (020 7436 5881)

Thank you very much for your help

Please return the questionnaire in the envelope provided. If you have any queries about the questionnaire or you would like to know more about the research please contact:

Maggie Redshaw or Rachel Rowe
National Perinatal Epidemiology Unit
University of Oxford
Old Road Campus
Headington
Oxford
OX3 7LF
Tel. 0808 252 4566

REC REF: 12/SC/0322 Q_N v3.3 101012

Sands
Stillbirth & neonatal death charity

Bliss
for babies born too soon, too small, too sick

PRUMHC
POLICY RESEARCH UNIT - MATERNAL HEALTH & CARE

npeu
National Perinatal Epidemiology Unit

UNIVERSITY OF OXFORD
